# Automatic assessment of the motor state of the Parkinson's disease patient--a case study

**DOI:** 10.1186/1746-1596-7-18

**Published:** 2012-02-19

**Authors:** Bozena Kostek, Katarzyna Kaszuba, Pawel Zwan, Piotr Robowski, Jaroslaw Slawek

**Affiliations:** 1 Multimedia Systems Department, Faculty of Electronics, Telecommunications and Informatics, Gdansk University of Technology, Gdansk, Poland; 2 Department of Neurology and Movement Disorders, St Adalbert Hospital, Gdańsk, Poland; 3 Department of Neurological-Psychiatric Nursing, Medical University of Gdansk, Gdansk, Poland

**Keywords:** Parkinson's disease, UPDRS, Rule-based decision algorithms, Rough sets

## Abstract

This paper presents a novel methodology in which the Unified Parkinson's Disease Rating Scale (UPDRS) data processed with a rule-based decision algorithm is used to predict the state of the Parkinson's Disease patients. The research was carried out to investigate whether the advancement of the Parkinson's Disease can be automatically assessed. For this purpose, past and current UPDRS data from 47 subjects were examined. The results show that, among other classifiers, the rough set-based decision algorithm turned out to be most suitable for such automatic assessment.

**Virtual slides:**

The virtual slide(s) for this article can be found here:

http://www.diagnosticpathology.diagnomx.eu/vs/1563339375633634.

## Background

The Parkinson's Disease is one of many neurodegenerative diseases which slowly degenerates the central nervous system. It results from lack of dopamine in brain cells, and most often manifests itself in motor complications. The causes of such disorders have not fully been recognized yet. Thus, medical treatment of PD patients is limited to reducing the disease symptoms. The progress of the disease is slow and may take several years. In its early stage, the illness is hard to recognize, however, when diagnosed it must be effectively treated to reduce its further development. Main PD symptoms are motor complications such as bradykinesia, muscle rigidity, freezing of gait, tremor [1], difficulties in swallowing, slow down or lack of facial expression and animation, etc. Beside causing motor disorders, PD may also impair concentration and daily routines planning.

Detecting the changes in PD patients state within short time is very difficult, in particular, because fully objective tests for evaluating the disease advancement have not been devised so far. One of the widely used methods to assess PD patients is conducting a series of normalized clinic tests known as the UPDRS--Unified Parkinson's Disease Rating Scale) [2,3], which is a list of 42 items with numbers assigned from a 0-4 range. The list is divided into 4 parts [2,3]. However, a subjective character of these tests introduces some error to their results.

Taken every three month or half a year, periodical UPDRS tests allow for the evaluation of the advancement of the disease. Such examination requires regular visits to specialists, and unfortunately this is often impossible to fulfill for PD patients for organizational reasons, for no access to specialists or simply because of deteriorating motor abilities. Other obstacles are problems with specifying the precise time of medications intakes or side effects of pharmacological therapy which cause dyskinesias (involuntary movement disorder). Some of late PD symptoms are consequences of long-term treatment with levodopa or dopamine receptor agonists [1]. At the beginning, levodopa considerably improves patients' condition and may help maintain such state for a number of years. With time passing, the effectiveness of levodopa diminishes and patients start experiencing "on" (normal) and "off" (with parkinsonian symptoms) states alternatively. The changes from "on" to "off" states are evidently related to medication intake schedule, and they are predictable for "on" states. Some patients may, however, experience abrupt changes to "off" states with no correlation to the time they took medicine, and additionally a so called on-off phenomena of rapid changes from "on" to "off" states may appear [1]. So, irregular or rare visits to physicians do not provide adequate information either on the advancement of the disease or on the appropriateness of the prescribed medication and its dosage. Above all, it is not possible to determine whether the patient is in the "on" or "off" state [1]. This excludes a full objective evaluation of a PD patient's state.

PD patients' ratings achieved through clinical trials often base on historical data from patients diaries. However, these data are in most cases subjective and not sufficiently precise. Thus, during recent years, an increased interest in patients' objective assessment has been observed, and several attempts to predict the state of PD patients have been made. In particular, many studies show that wearable sensors provide a way to record human activities continuously. Human activity recognition (HAR) plays an important role in health and elderly people care [4-6]. Not only the amount of movement but also precise information on its type is crucial in healthcare applications. Recognizing particular body activities let us detect disease symptoms, and analyze a patient's state [1,7-9]. Typically, 3-axis accelerometers are used in HAR to capture movement characteristics for different body positions [10-13]. The collected data can then be processed by different classifiers to recognize various activities of the monitored subjects. However, before applying any of the classification methods, a careful selection of pre-processing techniques and feature extraction methods should be performed.

A very valuable study by Patel et al. thoroughly reviews the state-of-the-art in the area of PD, and discusses limitations in PD patients' monitoring. The authors point out the main cause of limitations which for them is lack of integration between wearable technologies and algorithms used to estimate the severity of PD symptoms and motor complications. The paper by Patel et al. shows results of a pilot study on the feasibility of using data from wearable sensors to assess the severity of PD symptoms [1]. In this study, the authors used the data collected by a set of sensors in comparison with video recordings captured during the examination. The clinicians evaluated UPDRS scores based on video recordings, and compared them with estimates derived from the accelerometer data [1]. The paper also provides an exhaustive description of pre-processing and accelerometer-based feature extraction. The researchers used SVM (Support Vector Machine) with different configurations of settings and kernels as the classification algorithm. The paper by Patel et al. is one of the most important studies demonstrating that a continuous monitoring of PD patients can solve key problems in the assessment of PD progression. It also enables to estimate tremor, bradykinesia, and dyskinesia severity level [1].

Home monitoring of PD patients via wearable technologies and web-based applications is another study of remote objective long-term health monitoring [14]. Recently, the implementation of an iPhone estimating PD tremor with a wireless accelerometer application was also presented [15]. Initial testing and evaluation of this application successfully proves its capability to acquire tremor characteristics in autonomous environments [15]. A broader scope of healthcare application has a telemedicine instrument employed for remote evaluation of tremor: design and initial applications in fatigue and patients with Parkinson's Disease [16].

Speech degradation is one of the early symptoms of PD. Tsanas et al. [17] investigated tele-monitoring of PD progression by non-invasive speech tests. A methodology presented by Tsanas et al. is an example of mapping speech signal processing outcomes *(e.g. dysphonias *which are malfunctions in voice production) to predict clinical overview utilizing UPDRS metrics [18-21]. Two classification algorithms were employed in these studies*--*the *Classification and Regression Trees *(CART) and *Random Forests *(RF). Their studies proved that the classifiers can replicate the clinicians' UPDRS estimates with the accuracy considered sufficient for PD assessment. Moreover, they provide a statistical evidence that speech impairment and average overall PD symptom severity are inherently related. Thus, the approach based on speech processing and classification may be justified for UPDRS progression prediction [21].

A very promising approach was proposed by the PERFORM (A soPhisticatEd multi-paRametric system FOR the continuous effective assessment and Monitoring of motor status in Parkinson's disease and other neurodegenerative diseases progression and optimizing patients' quality of life) system, a European 7th FP project, that addressed this problem by designing and implementing a "Remote" Personal Health System [5,9]. The system objective was to continuously monitor patients in their homes by recording selected motor and non-motor parameters, and data from specific accelerometer sensors, and passing them to clinicians and specialists at Central Hospital Units. The methodology behind the system was to capture symptoms of PD and automatically assign UPDRS ratings [2,3] to them. After processing the data, PERFORM was supposed to generate alerts in the cases of emergencies [5].

**Figure 1:**
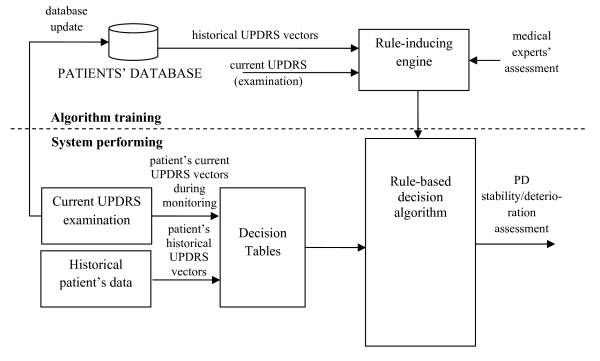
**General scheme of the automatic assessment of the PD disease deteriorating**.

This paper is a continuation of the previous work performed within the framework of the PERFORM project. It differs, however, in the approach to data acquisition, as no data from sensors are used (see Figure 1). Data come from patients' diaries and were rated by clinicians in the UPDRS scale. For the purpose of this study a computer application was prepared in Borland c++ environment. The training of the decision/support system was based on questionnaires used for classifying the motor state of the examined subjects. Decision tables were created and on this basis, a set of rules was generated taking into account historical and current UPDRS data from 47 subjects. This issue is described in the next Section. At the testing stage a decision algorithm that is incorporated into the system automatically assesses the patient's state. However to test overall efficiency of the decision system several algorithms were employed, first. Their efficiency and appropriateness for assessing the overall state of a PD patient are discussed in Section: Methods. Among tested algorithms the rough set-based approach was identified as the most efficient, and is applied in the methodology presented.

### Data

In order to obtain medical knowledge, historical UPDRS data of 47 patients from Saint Adalbert Hospital in Gdańsk, Poland were gathered. All trials and investigations have been approved by the Ethical Committee of the Medical University in Gdańsk, PL. Also consents in a written form from all patients in the UPDRS examinations were obtained.

Table 1 includes information on the subjects' sex and age. The subjects' average illness duration time was 9 (SD ± 5) years. The time period of historical UPDRS examination as compared to current examination was 8 months in average with the variance value of 7 months. For the assessment of PD progression, the UPDRS parts III and IV related to motor performance were used. Both historical and current evaluations were performed by clinicians. In the presented approach, the following 13 UPDRS items were assessed: UPDRS 13, 14, 20, 21, 23, 24, 25, 26, 28, 29, 31, 32, 39. Since PD is an asymmetrical disease, most of these symptoms are assessed separately for both right and left sides, resulting in 21 items.

**Table 1:** Average and variance statistics of the subject used in the experiment

subjects	number	average age	variance of age
All	47	68.2	9.8
Male	24	67.3	11.0
Female	23	69.1	8.6

**Figure 2:**
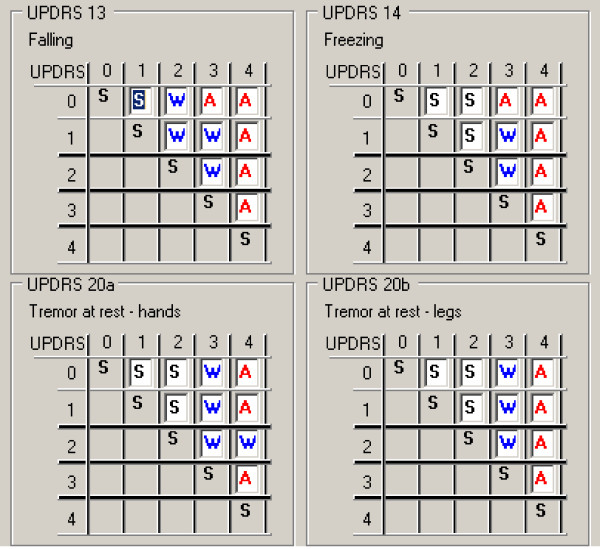
**An example of a decision table filled in by the clinicians**.

**Figure 3:**
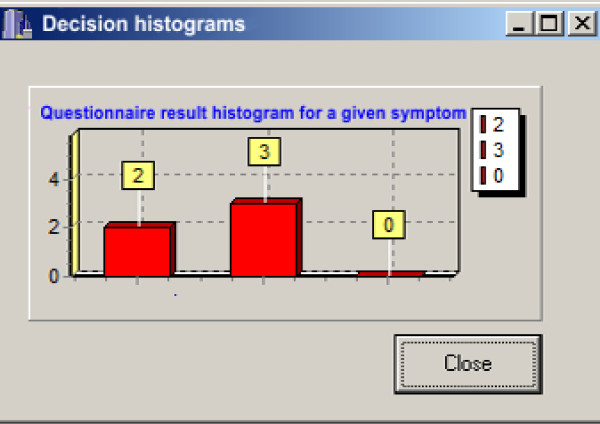
**Histogram related to a given decision table**.

Five experts (neurologists) participated in the creation of the decision tables, but only four of them evaluated the current patients' data. Their task was to assign a criterion--a decision attribute ("stable", "worsening", "alert") to every possible pair of the given UPDRS item. Changes from the higher to the lower score were not evaluated. For each record in the decision table a histogram of experts' decisions was created. Examples of such decision tables and a histogram are presented respectively in Figures 2 and 3. In the histograms information about number of experts voting for each criterion is presented (see Figure 3).

Since for 27 patients, an additional historical examination was available, in total 74 pairs of UPDRS evaluations between 'current UPDRS' and 'historical UPDRS' were used. As mentioned before the UPDRS pairs were assessed by 4 experts, thus the training set should consist in 296 training objects. However, eliminating superfluous data (entries in decision tables repeated) resulted in a reduced set of 284 elements.

## Methods

Several methods of automatic decision have now been implemented in today's medical applications, and presented in vast literature [22-24]. Especially interesting is a paper by Katsis et al., in which the authors describe a telemedicine platform with a decision system evaluating affective physiological states of the patients [25]. Most of the decision systems are learning algorithms, and some of them are rule-based, and can be easily interpreted by a physician.

In the application which automatically assesses PD patient state worsening, the following algorithms were compared: Rough Sets (RS) [26,27], generalized Rough Sets (RS-g) [28], the Repeated Incremental Pruning method (RIPPER) [29], the Nearest Neighbour algorithm (NN) [29], the PART Decision List with two sets of coefficients (PART) [29] and the Ripple Down Rule (RDR) method [30,31]. Particularly, the usefulness of rough sets needs to be stressed since this technique is widely used in data mining. Moreover, this approach seems very appropriate if the training set is of conflicting character, as happens in the case of assessment of PD patients' state worsening. Knowledge in this case is acquired through the analysis of questionnaires filled in by physicians, whose answers-depending on their experience and judgement--are to some extent subjective, thus may create conflicting data.

Since the rough set approach is less known than other learning algorithms, some basic information will be recalled here. Rough sets were introduced by Pawlak in the early 1980's [26]. They provide an effective tool for extracting knowledge from databases. Since that time many researchers applied this methodology in various areas [32-34]. A fundamental principle of a rough set-based learning system is to discover redundancies and dependencies between features describing a problem to be classified. A data set is represented as a table, where each row is a case, an event, a patient, or simply an object. Every column is an attribute (a variable, an observation, a property, etc.) that can be measured or provided by a human expert or user for each object. This table is called an information system [32]. In many applications the outcome of the classification is known. This a posteriori knowledge is expressed by a special attribute called a "decision attribute". Information systems of this kind are called decision systems. It is assumed that a decision system (i.e. a decision table) expresses all the knowledge about the model. This table may grow unnecessarily large because it is redundant in at least two ways. Either indiscernible objects may be represented several times, or some of the attributes may be superfluous. In a classical set theory, every element either belongs to a set or it does not. The corresponding membership function takes 1 or 0 values, respectively. In the case of rough sets, the notion of membership is different. The rough membership function quantifies the degree of a relative overlap between set X and the equivalence [x]_B _class to which x belongs. There are many properties of rough sets. A *Universe *is defined as a collection of objects standing at the top of the rough set hierarchy. On the other hand, a basic entity is placed at the bottom of this hierarchy. Between them, the *Approximation Space *is defined. The *Approximation Space *is partitioned by the minimum units, called equivalence classes, or elementary sets. Lower and upper approximation definitions are based on the approximation space. Consequently, a *rough set *approximates a given concept from below and from above, using both lower and upper approximations. Other properties of rough sets are a reduct and a core. However, it should be pointed out that the rough set-base algorithm is not the only one method of dealing with uncertainty [32-34].

As mentioned in Introduction the scheme of the presented methodology is shown in Figure 1. In our approach, the automatic assessment of the stability/worsening of PD patients is based on the comparison of current and historical UPDRS rates stored in a database. In this case, a physician's decision is needed to obtain knowledge of how to translate changes of the UPDRS scores into stability/deteriorating assessment. These pairs of UPDRS vectors were presented to four medical doctors and each of them assessed the state of the patient by using a decision scale: "0"-no deteriorating (stable), '1'-slight deteriorating (worsening), "2"-severe deteriorating (alert). Definitions of "slight" and "severe" had been earlier discussed with the neurologists and related to the alert level that should eventually be raised. Slight deteriorating should raise a low priority alert (warning) while severe deteriorating should cause a high priority alert (alarm). Each of the UPDRS pairs was assigned to three output classes: 0, 1, 2, and therefore the obtained data could have been used to train decision systems. Neurologists were instructed not only to sum up the UPDRS points, but also to consider the importance of each of the symptoms when assessing the alert. The rules obtained in the training stage are then used by the support system, which compares UPDRS scores assessed automatically with historical ones from the database. As a result an automatic evaluation of improvement/deterioration is generated for a monitored PD subject.

All data were divided into training and test sets in a 50:50 proportion, with 142 training objects contained in each set. Classifiers based on rules extracted for the training set were then tested. The test set was used to verify the generalization qualities of the classifiers.

**Figure 4:**
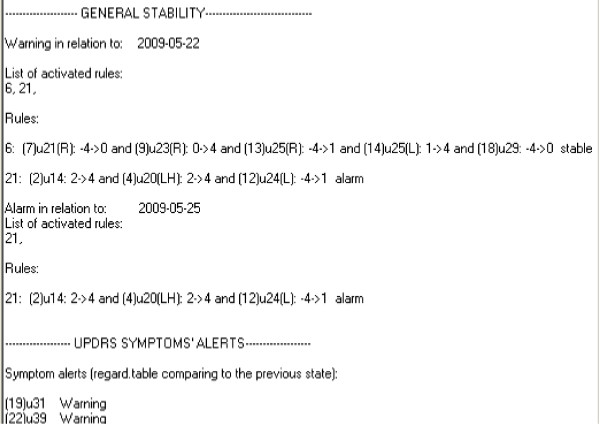
**An example of a report generated by the computer application prepared for this study**.

An example of a rule calculated by the rough set algorithm is presented below:

$$\begin{array}{c} If\left(\Delta UPDRS13<1\right)\&\left(-1<\Delta UPDRS14<2\right)\&\left(\Delta UPDRS23RH<1\right)\&\\ \left(\Delta UPDRS29<1\right)=>\left(\left\{output=1{-}^{'}warning^{'}\right\}\right), \end{array}$$

and also in a report from the computer application (see Figure 4).

The rule antecedent prepositions are the changes in UPDRS ratings for a given symptom. This rule gives information which UPDRS values showed critical differences in the ratings. In this context rules can be easily interpreted by a doctor.

## Results

**Figure 5:**
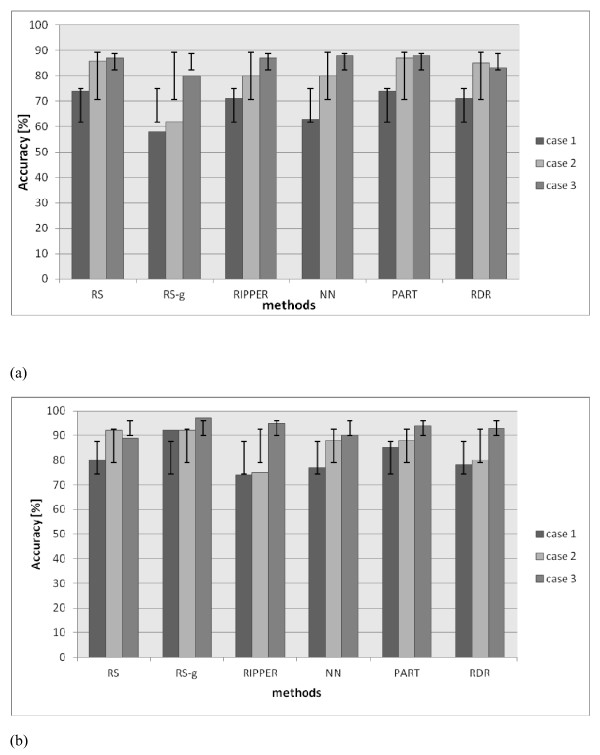
**Results of classification for training (a) and testing (b) data**.

The algorithms generated the following number of rules, i.e. RS--240 rules, RS-g--180 rules, PART--17 rules, RDR--13 rules, NN--34 rules, RIPPER--5 rules. The efficiency of each classifier was tested first on the training set and then on testing data. In the first case, the ability to cover the training data efficiently was tested, in the second the generalization quality of the classifiers was investigated. The efficiency of the classification was calculated using a confusion matrix, i.e. a table presenting separately the recognition results for each of the recognized categories. Three types of the classification efficiency were defined: case 1: the efficiency of 3 classes recognition: {0,1,2}, case 2: the efficiency of the recognition of a high priority alarm {{0}∪{1}, {2}} and case 3: the efficiency of the recognition of any alert {{0},{1}∪{2}}. The results of the classification of training and testing data for these three cases are presented in Figure 5.

Since data coming from subjective evaluation may be inconsistent, a 100% accuracy cannot be achieved. The maximum accuracy achieved by this system is related to the coverage of the training examples. The highest values are 74% for case 1; 86% for case 2; and 87% for case 3. In all cases, the best accuracy has been achieved by the RS classifier.

## Discussion

The best results are achieved by the rough sets-based classifier. The drawback is the loss of the accuracy for data from outside the training set. If a generalization algorithm is additionally applied to RS it improves the accuracy making it close to the accuracy obtained for the training data (without generalization). The achieved accuracy is 68% in the first case, 79% in the second case, and 84% in the third case. The remaining four algorithms show worse results. Only for the PART decision list, the accuracy is similar to the RS algorithm for recognizing training data. Conversely, the recognition of testing data is much worse. The set of rules for PART consists of 17 rules only, so their analysis is easier for the doctors but the classifier is performing well only for the training data without any generalization capacities. Due to this, the rough sets algorithm, has been chosen for the automatic assessment of the PD patients' state deterioration. It is based on the information acquired from sensors and the UPDRS examination results. In addition, the generalization quality of the system implies that it attempts to mimic doctors' reasoning.

## Conclusions

The presented methodology of automatic assessment performed for the motor state of PD patients seems to be valuable. Since neurologists who took part in this study have been instructed not only to sum up the UPDRS points, but also to consider the importance of each of the symptoms when assessing the alert, thus in this sense, the rules acquired from the learning algorithm reflect the importance of each of the UPDRS inputs in the overall evaluation. The amount of the collected data may be increased, and then the system may easily be retrained. This may result in better overall accuracy and may be valuable for a medical doctor who is not a neurologist.

## Abbreviations

UPDRS: Unified Parkinson's Disease Rating Scale; RS: Rough sets; RS-g: Generalized rough sets; RIPPER: Repeated incremental pruning method; NN: Nearest neighbour algorithm; PART: PART decision list with two sets of coefficients; RDR: Ripple down rule.

## Competing interests

The authors declare that they have no competing interests.

## Authors' contributions

Conceived and designed the experiments: BK, PZ, JS. Performed the experiments: PZ, KK, PR. Analyzed the data: JS. Wrote the paper: PZ, BK. Revised the paper: JS, BK. All authors read and approved the final manuscript.
